# Medical and Philosophical Intelligence

**Published:** 1818-06

**Authors:** 


### MEDICAL AND PHILOSOPHICAL INTELLIGENCE.

*To the Editors of the London Medical and Physical Journal.*

GENTLEMEN,

A CASE of Peritonitis has recently occurred in the person of Mrs. G. of Beech-street, two weeks after delivery, with all the customary symptoms of that frequently unmanageable malady. The ordinary antiphlogistic plan was actively pursued, without much apparent advantage, for two days, when (from a suggestion given by Dr. Blegborough) I ordered a quart of warm milk to be thrown up the bowels as a fomentation: this had been done only ten minutes, when the uneasiness in the abdomen began to cease, and, in ten minutes more, had ceased altogether; and convalescence rapidly and completely supervened. I remain, your's, &c.

*Queen-street, Cheapside.* ED. SUTLIFFE.

---

*Royal Society.*—April 2. A paper, by Mr. JOSEPH SWAN, was read, giving an account of a new method of making anatomical preparations.

On the same evening, a paper, by Dr. JOHN DAVY, was read, on the urinary organs and secretions of some of the amphibia. In several species of serpents, which were examined by the author, the kidneys were found of very considerable size, and of a long and very narrow form. Ducts proceeded from them to the ureters, which last terminate in what appeared to be a distinct receptacle, communicating with the rectum by a sphincter. A white matter is deposited in the urinary passages, which is occasionally expelled by a kind of extraordinary effort, and which consists of uric acid. The urinary organs and secretions of lizards were found to be nearly similar to those of serpents. The fluid appears to contain no urea.

April 9.—A very important paper, by Sir H. DAVY, was read, containing an account of a series of experiments on the combinations of phosphorus with oxygen and chlorine. [The further notice of this we reserve till the whole appears, as doubtless it will, in the Transactions of the Society.]

April 16.—A paper, by Dr. GRANVILLE, was read, on a particular malconformation of the uterine system in women, and on some physiological conclusions to be deduced from it.

The case consisted of a female whose uterus was found after death to have been entirely imperfect on one side, and to have had one set only of the lateral appendages; yet she had been the mother of eleven children, some of each sex, and was delivered of a boy and a girl at one birth. This case completely proves the fallacy of a physiological hypothesis, which has been proposed, that the two sexes are formed on separate sides of the uterine system.

---

*Royal Academy of Sciences at Paris.*—Jan. 5, 1818. M. VAUQUELIN was nominated vice-president.

M. FOUCIER, in the name of a committee, read a report on a prize to be offered on the subject of Statistics.

Dr. THOMAS YOUNG, secretary of the Royal Society of London, was elected a corresponding member in the section of natural philosophy.

Committees were appointed by ballot to examine the memoirs of the several candidates for the prizes offered in natural philosophy, mathematics, and chemistry.

Jan. 12.—Committees were nominated to draw up reports on the subjects and conditions of the prizes for next year, and to adjudge the medal of Lalande.

M. Percy made a report on several works and collections relative to medicine.

Jan. 19.—M. Pons, *directeur-adjoint* of the observatory of Marseilles, announced that he had discovered a new comet.

Jan. 26.—The minister of the interior requested the academy to nominate a candidate for the situation of *second professeur adjoint* in the School of Pharmacy at Montpellier.

M. Devaux gave in a memoir on the glands of vegetables.

M. Vauquelin read a memoir on the influence of the metals in the production of potassium by means of charcoal.

Feb. 2.—M. Percy delivered in the report of a committee on a memoir by M. Roux, on the operation for the cataract.

The object of this paper is a comparison of the two methods at present in use of operating for the cataract, namely, by extraction or depression. He observes first, that each method has been long practised; Philoxenus, 270 years before the Christian era, having operated by depression; and Antylus having employed the method by extraction near the end of the first century. M. Roux is an advocate for the method by extraction, and states that out of 660 operations performed by him, for the most part on hospital patients, 400 have been attended with success. The committee, in their report on this paper, state that, out of 65 operations performed at the Hotel Dieu by the method of depression, 48 have completely succeeded. Hence they conclude that a rational practitioner will not bigotedly confine himself to either of the two methods, but will be determined in each case by particular circumstances.

In compliance with the invitation of the Minister of the Interior, the section of chemistry recommended M. Duportal for the office of *professeur-adjoint* to the School of Pharmacy at Montpellier.

Feb. 9.—M. Percy, in the name of a committee, made a report on the medical properties of the preparations of gold.

Dr. Chrestien, of Montpellier, has of late exerted himself to restore to medicine the preparations of gold, which, although highly extolled by the older physicians, have wholly disappeared from modern pharmacopœias; the committee, in order to qualify themselves to form an accurate judgment on the subject, have made experiments, from which they conclude that the salts and other preparations of gold have a decided action on the animal economy, which it is well worth the while of medical practitioners carefully to investigate. They have themselves ascertained that friction of the tongue and gums, with four grains of powder of gold, will (as stated by Dr. C.) produce sometimes a copious ptyalism, sometimes abundant alvine evacuations, and sometimes profuse perspiration.

M. Risso presented a memoir, entitled "*Coup d'œil geologique sur les Environs de Nice.*"

M. Morichini writes word that his experiments on the magnetic power of the violet rays continue to be attended with success.

M. Cuvier communicated some observations on several sculls of the ourang-outang, from which he concludes that the East Indian animals hitherto described under this name are probably only young specimens of the large species of monkey, called Pongo by Wurmb.

Feb. 23.—M. Dumeril read a report on certain pieces of apparatus, presented by M. Brizé-Fradin, for the purpose of purifying foul air.

---

Many alterations in the Court of the *Royal College of Surgeons in London* having taken place since the last list was published, we give the following as a correct account of the present members.

*Master.*—George Chandler, Stamford-street.

*Governors.*—Thomas Keate, Surgeon to the Queen and Prince Regent, Chelsea; David Dundas, Serjeant Surgeon to the King, Richmond.

*Assistants.*—*Thompson Forster, Southampton-street; *Sir Everard Home, Bt. Serjeant Surgeon to the King, Sackville-street; *Henry Cline, Lincoln's-inn-fields; *William Norris, Old Jewry; John Heaviside, Surgeon Extraordinary to the King, George-street, Hanover-square; *Sir William Blizard, Devonshire-square; John Adair Hawkins, Great Marlborough-street; Francis Knight, Saville-row; *Sir Ludford Hervey, Kt. Red Lion-square; *William Lynn, Parliament-street; John Abernethy, Bedford-row; William Lucas, Threadneedle-street; Astley P. Cooper, Spring-gardens; Anthony Carlisle, Surgeon Extraordinary to the Prince Regent, Soho-square; Thomas Chevalier, Surgeon Extra to the Prince Regent, South Audley-street; James Wilson, George-street, Hanover-square; John Gunning, Surgeon Extraordinary to the King, Lower Grosvenor-street.—(*Those marked thus * are Examiners.*)

---

The *Medical Society of London* adjourned in the middle of May, on account of improvements to be made in their house, particularly in the arrangement of their valuable library.

---

We extract the following as part of the luminous annual Report of the illustrious Cuvier: most of the other particulars have appeared in our Journal, as they were read before the Institute.

After passing in review the labours of M. Magendie and M. Pelletics on *Emetine*, he stated those of M. Robiquet and Orfila on *Morphine*, a principle contained in opium, and which, by its union with an acid called *meconic*, contained in the same substance, appears to be the really narcotic part of opium.

He afterwards passed to the experiments of Dr. Edwards, on the asphyxia of the *Batræcians*, and of which the principal result is, that frogs, toads, and salamanders, from which the heart had been removed, and in which consequently all circulation and pulmonary respiration was suppressed, lived longer in the open air than in water, or water deprived of air; from whence it results, that, in these experiments, ***air has on vitality an influence independant of the lungs and the circulation.***

---

We pass over M. Mejendie's theory of the arterial structure, as requiring further experiments. At present, they proceed no further than was explained by Dr. Young some years ago in the Philosophical Transactions of London. The result of all the cruel experiments on the process of vomiting, we have before remarked, have been by Mr. Hunter demonstrated without the necessity of instituting any experiments directed immediately to that object. A few of MM. Portal's and Gerard's remarks are worth transcribing.

M. Portal, in a memoir on vomiting, after having cited the former experiments he had made, and in which, after cutting the muscles of the lower belly, he saw the stomach expand and contract with force when the diaphragm was thrown back on the breast, shewed the manner in which he conceived that the rejection of food is produced.

M. Girard, Director and Professor of Anatomy in the Veterinary School at Alfort, has presented a paper on vomiting, as considered in different domestic animals. In general, the more the œsophagus in the cardia is towards the left, the more it widens the weaker and fleshy fibres which surround it; and the more the grand cul de sac is effaced, the more the pylorus is contracted; and the more the veil of the palate is moveable and drawn up, the easier the vomiting is. It is then much so in carnivorous animals, in which the stomach is little more than an oblique distension of the alimentary canal.

It is painful in the pig, where the bag on the left takes up half the place of the viscera, and where the œsophagus is very narrow, and lined with a thick fleshy layer. In the horse, when the stomach, at a distance from the muscles in the abdomen, is, besides the cardiac, near the pylorus, crossing the coats obliquely, and strongly surrounded by fleshy layers, the vomiting does not take place in a natural state. It is still more rare in ruminating animals, on account of the complication of the four stomachs, and the singular manner in which the œsophagus joins them; but such vomiting may occur contrary to nature, and which is frequently followed by death.

*** Horses are generally supposed never to vomit, but at sea they often do. Eng. Editor.

---

At length the two Royal Colleges are beginning to show marks of industry, which, we doubt not, will exhibit what was once considered the temple of Morpheus in some state of activity, and render the new Royal Establishment worthy of its honourable title, and of the valuable treasure it possesses in the accumulated industry of Mr. Hunter, and several others.

In Warwick-lane, (a sound almost ridiculous since the days of Foot,) the three sets, we cannot call them courses, of lectures, have commenced under the happiest auspices. Dr. Tuthill, on poisons, has given three lectures, completely classical, historical, geographical, chemical, and pathological; in a word, they were worthy of being listened to by the learned audience in whose presence they were pronounced. It appears, that the lecturer was present at Paris when several of the experiments were made, in some of which Mr. Brodie was anticipated. The course concluded with an experiment on a living guinea-pig; by which it was shewn that the action of the heart might be continued when respiration had ceased. The Gulstonian Lectures were commenced by Dr. Powell. These relate to the same subject as those cases, by the same gentleman, which we extracted from the last volume of the College Transactions. In the lectures already delivered, a number of valuable cases of extravasations in the brain are minutely related, partly from the lecturer's own observation, and partly from other writers. The inference from the whole has not yet been delivered, but we doubt not it will lead to some important improvements in the pathology of that ill-understood organ.

---

At the *College of Surgeons* Mr. Carlisle has commenced a course of lectures on surgery, chiefly such as might be expected, when delivered to an audience already acquainted with the subject. This circumstance seems to have somewhat embarrassed the lecturer, who every now and then appears at a loss, *quid dem quid non dem?* as if occasionally viewing the younger part of his audience at a distance, and, at other times, those in the benches around him. Mr. Lawrence is to succeed him on comparative anatomy.

The Museum is exhibited two days in every week till the conclusion of August.

---

Division of vena saphena, on a subject 45 years old, was performed, a few days since, at one of the public hospitals according to the mode recommended by Mr. Brodie. The operation was completely successful, and no ill consequences followed.

---

A case of ulceration in the lower lip of a man, upwards of fifty years old, which, in the opinion of one of the ablest surgeons at a royal hospital, had many characters of cancer, has been cured by the application of the white oxide of arsenic. The ulceration had destroyed the greater part of the lower lip, and (which rendered the prospect more gloomy,) the patient's father had died of a similar disease. The arsenic was applied till the morbid part was entirely destroyed; after which the part healed readily.

*** When shall we see the Reports of the late Dr. Denman's Cancer Institution?

---

A man in St. George's Hospital has lost the sight of one eye. The anterior chamber is filled with coagulated lymph, organised, as appears, by a cluster of blood vessels, which may be discovered over the part which was once the pupil. The cornea and conjunctiva retain their natural appearance. It is not fair to trust to the imperfect accounts of patients: but his history of the disease is, that high inflammation occurred eighteen months ago, for which leeches and a blister were applied; in about a fortnight's time, his eye was irrecoverably lost. He had not used mercury that he knows of, nor had any venereal symptoms for sixteen years before.

He has been in the hospital five months, using mercury apparently with little if any advantage.

We have lately congratulated our brethren on the prospect of curing tic douloureux by means of belladonna. We are now informed that, when the disease is become chronic, it may be relieved by the local application of tar. Some impute the whole benefit to the superficial inflammation excited by the remedy; but it must be admitted that other rubefacients, and even vesicatories, have been less successful.

---

The *hermaphrodite*, which we mentioned nearly two years ago, as exhibiting itself in Paris, has now been long enough in London to satisfy the curiosity of many. For the benefit, however, of our country friends, and of such Londoners as have not found their way to an unadvertised spectacle, we subjoin a few words. The figure is about the middle height, and completely Grecian; the countenance scarcely less so; complexion dark, but clear; beard very general and thick on the chin and upper lip, but only on the latter of any length; a considerable part of the skin, also, as hairy as in men; voice feminine; hips broad; *pectora fœminea et optime formata; penis dictus, nihil nisi clitoris longissima; vagina integumento communi occlusa prœter foramina duo per quœ urina transit; catameniœ, plurimum regulares. Lutitiœ, dicitur viros petiise, nunc pro fœminis appetentiam quasi effrœnatam profetetur. Nobis in dubio est si unius vel alterius desiderium sentit, quemadmodis in brutis, dubii generis, cupido omnis coeundi abest, neque petuntur a maribus vel fœminis.*

---

Trials have been frequently made, but without success, to multiply the olive by sowing the seeds; it has always been found necessary either to employ cuttings, or to procure wild plants from the woods. One of the inhabitants of Marseilles, astonished to find that we cannot obtain by cultivation what Nature produces spontaneously, was led to reflect upon the manner in which the wild plants were produced. They proceed from the kernels, which kernels have been carried into the woods, and sown there by birds, who have swallowed the olives. By the act of digestion, these olives have been deprived of their natural oil, and the kernels have become permeable to the moisture of the earth, the dung of the birds has served for manure, and, perhaps, the soda which this dung contains, by combining with a portion of the oil which has escaped digestion, may also favour germination. From these considerations the following experiments were made.

A number of turkeys were caused to swallow ripe olives; the dung was collected, containing the kernel of these olives, the whole was placed in a stratum of earth, and was frequently watered. The kernels were found to vegetate, and a number of young plants were procured. In order to produce upon olives an effect similar to that which they experienced from the digestive power of the stomach, a quantity of them was macerated in an alcaline lixivium; they were then sown, and olive plants were produced from them as in the former experiment.

This ingenious process may be regarded as a very important discovery, and may be applied to other seeds besides that of the olive, which are, in the same manner, so oily, that, except under some rare circumstances, the water cannot penetrate them and cause their development. Of this description is the nutmeg, which will seldom vegetate in our stoves; but which, perhaps, would do so, was it submitted to the action of the stomach, or of the alkaline solution.

[It does not seem to be within the knowledge of our neighbours that the seed of the hawthorn, which usually requires two years before it shoots above the earth, may be hastened a whole season by being swallowed by cattle. Instead of lixivium, we conceive gastric juice would be the proper substance. This discovery of the hawthorn-seed is due to Sir Joseph Banks.

---

The following we copy from the *Annals of Philosophy.*
(*To Dr. Thomson.*)

Sir,—The powers and facilities of chemical analysis have been of late much improved. Heat and chemical mixture being the great agents in this analysis, whatever augments those powers, or facilitates their application, must also aid in effecting this process. The powers of the solar burning lens, or mirror, had long ago produced effects far beyond the reach of fuel and furnaces. The expensive nature of this apparatus, however, limited its use, and chemists had to regret that the same zeal and munificence which had been employed to extend the powers of the telescope, had not been also employed to furnish a chemical instrument of analogous power in the solar lens and mirror.

The powers of galvanism, as an instrument or aid in analysis, left little room for regret in this respect. The alkalies and several of the earths were decomposed, and their metallic bases discovered. This power, however, is both expensive and of limited application. But, though galvanism is so expensive, and capable of operating on only small quantities of matter, its successful application to chemical analysis was deservedly hailed as marking an era in science, and celebrated over the civilized world.

So rapid, however, is the progress of improvement, that the late discovery or revival of the use of a mixture of those gases which contain the ingredients of water, applied by the blow-pipe to promote combustion, is likely to supersede the employment of galvanism in chemical operations. The degree of heat thus obtained, is sufficient to fuse or resolve substances the most refractory, and the application of a blow-pipe is a simple manipulation.

The great difficulty in the use of this gaseous mixture arises from its very explosive qualities; and the hazard of the ignition passing backward along the tube of the blow-pipe to the magazine. From the combustible nature of the mixture, it should seem wonderful that it could have been used once without explosion. Perhaps, it was owing to the rapidity of the gas issuing, exceeding the velocity with which the ignition could pass against the direction of the current backwards to the gasometer. If so, the danger must increase as the gasometer becomes exhausted, and as the issuing current becomes less rapid.

To obviate the danger arising from this tendency of the gaseous mixture to explode, various contrivances have been adopted. Some, to secure against danger in case of explosion taking place; some, to prevent its occurrence. The latter object is certainly the most important.

This object has been attempted to be gained by interposing a fluid between the lamp and gasometer. A partial explosion, however, might agitate the fluid, or displace a quantity of it, and occasion inconvenience or danger. The addition of the principle of the safety-lamp seems the most promising expedient. The wire-gauze lamp prevents explosion, by cooling the gas down below the exploding heat, as it passes the meshes of the gauze. To gain the same end, in the use of the gas blow-pipe, one philosopher has proposed a fagot, or bundle of small tubes, to be enclosed in a larger tube, and the gas to pass through these. Another has proposed to enclose, in the tube through which the gas passes, a number of folds of wire-gauze. Either of these expedients seems capable of affording the desired security, if the plan be perfectly executed.

But a still more simple and facile apparatus presents itself. It is to make a pretty long portion of the tube through which the gas passes, sufficiently wide, and to enclose in it, instead of a bundle of tubes, or a quantity of wire cloth, a bundle of wires placed longitudinally. The tubes formed by the interstices of these wires are of a much better form, and in proportion to their capacity present a much greater cooling surface, than cylindrical tubes. The apparatus is of the easiest construction. Its powers, as a safe-guard, may be almost indefinitely augmented. The wires may be used very fine, and, perhaps, may be advantageously twisted; the fagot of wires may be increased at pleasure, both in number of threads and in their length; and, in each of these ways, or by them all combined, the powers of this apparatus may be augmented.

The same principle may be applied in various other forms; *e. g.* one of two polished metallic plates might be furrowed with intersecting lines. The plates might then be applied to each other, and their edges soldered or cemented together, except at two points; one for the entrance, and another for the exit, of the gas. These plates, thus applied, would form a part of the passage between the lamp and gasometer. This too is capable of augmentation at pleasure. Both this and the tube with wires might be easily surrounded, when in use, with cooling or freezing mixtures. By such means, the powerful aid of the gas blow-pipe may be obtained with a great degree of safety.

*Glasgow;* I am, Sir, your obedient servant,

*Nov.* 20, 1817. JAMES WATT, M.D.

A good deal has been lately said upon the deterioration of the climate of this country. We have been anxious to obtain facts, so as to endeavour, if possible, to set the question at rest. We find, upon reference to the Meteorological Journals of the Philosophical Transactions, that for the last fifty years the average difference in the heat, for the whole year, does not vary three degrees; it having fluctuated between 51° and 49° of Fahrenheit. But, upon comparing the heat of the five summer months, for the years mentioned below, it will be seen that there is a very material difference: we have taken as far as the journals are correct, every fifth year:—

| | May. | June. | July. | Aug. | Sept. |
|---|---|---|---|---|---|
| 1775 | 74.5 | 81.5 | 82 | 75.5 | 75 |
| 1780 | 84.5 | 84.5 | 82 | 53.5 | 84 |
| 1790 | 66 | 86 | 73.5 | 77 | 68 |
| 1795 | 81.5 | 77.5 | 76 | 79 | 78 |
| 1800 | 75 | 75 | 81 | 89 | 77 |
| 1805 | 72 | 75 | 79 | 78 | 80 |
| 1810 | 67 | 78 | 78 | 80 | — |
| 1815 | 68 | 70 | 72 | 69 | 70 |

These numbers are the maxima of the heat of the respective months —*Monthly Mag.*

It is to be regretted that we have no authentic diaries earlier than the above period.

*Case of a Foreign Substance lodging in the Lungs;* by J. B. BROWN, M.D.—C. B. the son of a gentleman of this town (Boston), thirteen years old, swallowed a white tack, or pump nail, Feb. 1814. Soon after, he had a slight cough and occasional difficulty of breathing, which was always imputed to a cold, and excited so little alarm, that his parents did not consult a physician until March 1817. I was then sent for, and found the lad apparently attacked with pneumonia. He was bled and blistered, which gave him relief, and, in the course of three weeks, he was able to return to his school in the country, where he had been for nearly two years. About the latter part of April, he sent word to his parents, that he had got rid of the tack, and, at the commencement of his vacation in May, he brought it home with him. The account he gave me is this: that he raised some blood, soon after which, he had a fit of coughing; and, feeling something hard in his mouth, he, with his fingers, took out this nail or tack. I examined it, and found about one-third of it, in the state of a black oxyde, which was separated from the rest by handling. The remaining part of the nail retained its usual form, but was discoloured. Since this occurrence, I have been told of a similar accident that happened to a child in the town of Wilmington, Massachusetts. It swallowed a melon-seed; and, thirteen months after, in a violent paroxysm of coughing, its mother gave it a slap on the back, and the seed was forcibly expelled from the mouth. This child was afflicted with a cough and affection of the chest, during the retention of the seed, which entirely left it after its ejection. I find in the New-England Journal, vol. ii. p. 89, an account, which John Mervin Nooth, M.D. F.R.S. gives of his own case.—He swallowed a leaden shot, in the last glass of a bottle of wine, and ejected it thirteen years afterwards, in a paroxysm of coughing. For the two last years he laboured under a violent affection of the chest, which, at length, had arisen to such a height as to threaten immediate death. His symptoms were alleviated by the ejection of the shot, and he soon got well.

From the consideration of the above cases, is it not natural to conclude, that phthisis pulmonalis arises frequently from foreign substances, producing irritation, inflammation, and, finally ulceration of the lungs?—There can be little doubt, that these cases would all have terminated fatally, had not the substances been fortunately expelled.—*New-Eng. Journ.*

---

[From the same Journal we extract the following case: two such have lately appeared in our Journal, we conceived, sufficiently authenticated, but are informed the success of the operation is still doubted by some.]

In the fifth volume of your Journal, I communicated a case of diseased foot, with an account of the amputation of a part of it, by sawing through the bones. I stated at that time, that the cure was, probably, retarded by the disease having been suffered to continue so long, and that I had but little doubt, that, if the same operation was performed, where the disease was recent, that the parts would speedily heal. Since that time, I have had an opportunity of performing the operation, precisely in the same way, excepting that the metatarsal bones were sawed through, instead of the cuneiform. The patient was nearly sixty years of age, and addicted to an irregular and intemperate mode of life. The disease, however, which originated from frost, was recent, of only a few weeks standing. For the first three days succeeding the operation, the appearances of the stump were unfavourable, and the discharge consisted of an offensive bloody ichor; there was, also, some sloughing of the integuments. A liberal use of wine and bark in substance, produced a healthy discharge, and, in seven weeks, the stump was healed, so that the patient could walk on it with great ease. At that time, a very small opening was made in it, which lasted two or three weeks, and was occasioned by the patient's striking it in walking incautiously. There were no unpleasant symptoms, and no appearance of exfoliation during the cure, and that foot is now nearly, if not quite, as useful as the other. As this operation can be performed so much more expeditiously than that of disarticulation, and, in many cases where the disease will not admit of removal at the joints, and as it can be done, with so much more ease to the patient as well as the surgeon, I can see no reason why it should not, in every case, be substituted for it. GEO. HAYWARD.

*Note.*—The English surgeons usually practise the operation of sawing the bones of the foot, while the French prefer separating them at their articulations. The former operation is the most easy to be performed, the latter is the least liable to be followed by exfoliation.—EDIT.

---

*On the Magnetizing Power of the Violet Rays of the Solar Spectrum.*—The reported discovery of M. Morichini, respecting the magnetizing power of the violet rays, which was scarcely credited in this country, has received the confirmation of Professor PLAYFAIR, as related in one of the late numbers of the *Bibliotheque Universelle.* He gives the following account of an experiment of which he was a witness, and which was performed by M. Carpe.

After having received into my chamber a solar ray through a circular opening made in the shutter, the ray was made to fall upon a prism, such as those which are usually employed in experiments upon the primitive colours. The spectrum which resulted from the refraction was received upon a screen; all the rays were intercepted, except the violet, in which was placed a needle for the purpose of being magnetized. It was a plate of thin steel, selected from a number of others, and which, upon making the trial, was found to possess no polarity, and not to exhibit any attraction for iron filings. It was fixed horizontally on the support by means of wax, and in such a direction as to cut the magnetic meridian nearly at right angles. By a lens of a sufficient size, the whole of the violet ray was collected into a focus, which was carried slowly along the needle, proceeding from the centre towards one of the extremities, and always the same extremity, taking care, as is the case in the common operation of magnetizing, never to go back in the opposite direction. After operating in this manner for half an hour, the needle was examined; but it was not found either to have acquired polarity, or a sensible attraction for iron filings. The process was then continued for twenty-five minutes more, (fifty-five in the whole,) when the needle was found to be strongly magnetic; it acted powerfully on the compass, the end of the needle which had received the influence of the violet ray repelling the north pole, and the whole of it attracting and keeping suspended a fringe of iron filings.

It is stated, that a clear and bright atmosphere is essential to the success of the experiment, but that the temperature is indifferent. At the time when the above experiment was made, about the end of April, the temperature was rather cool than warm.—*Annals of Philosophy.*

---

*Adv.*]—*Just received from Paris*, in imperial quarto, illustrated by twenty-three highly finished engravings, beautifully coloured, "Nosologie Naturelle, ou les Maladies du Corps Humain distribuées par Familles; par J. L. Alibert, Chevalier de Plusieurs Ordres, Medecin Consultant du Roi, &c. &c. Tome premier."—To be had of J. Souter, 73, St. Paul's Church-yard.

---

In the press, Observations on the Properties of the Air-pump Vapour-bath, pointing out their Efficacy in the Cure of Gout, Rheumatism, Palsy, &c.; with Cursory Remarks on Factitious Airs, and on the improved State of Medical Electricity, in all its Branches, particularly in that of Galvanism, and their Efficacy in various Diseases. By M. La Beaume, Medical Electrician.

Shortly will be published, Observations on a Stridulous Affection of the Bowels, and on some Varieties of Spinal Disease; with an Appendix of Cases. By J. Bradley, M.D.

Dr. Bateman is preparing for the press, A Sketch of the Character of the Epidemic Fever prevailing in the Metropolis, with some Observations on the Method of Treatment, and on the Means of diminishing the Influence of Contagions.

---

*Adv.*]—Dr. Davis will commence a Course of Lectures on the Theory and Practice of Midwifery, and on the Diseases of Women and Children; on Monday the 8th of June, at his house, No. 29, George-street, Hanover-square, at half-past ten o'clock in the morning.

---

*Adv.*]—Dr. Merriman, Physician-Accoucheur to the Middlesex Hospital, and Dr. Ley, Physician-Accoucheur to the Westminster Lying-in Hospital, will commence a Course of Lectures on the Theory and Practice of Midwifery, and the Diseases of Women and Children; on Monday the 1st of June, at half-past ten o'clock. —Particulars may be learnt at Dr. Merriman's house, 26, Half-Moon-street, Piccadilly; at Dr. Ley's, 62, South Audley-street, Grosvenor-square; and at the Middlesex Hospital, where the lectures will be given.

---

Dr. Bostock proposes to give a Course of Lectures on Physiology and Animal Chemistry, during the next winter.

Dr. John B. Davis commenced, on the 18th of May, Lectures on that Branch of the Practice of Medicine which relates to the Diseases, Medicinal Management, and Nursing, of Children.

---

### REPORT OF DISEASES.

We have but little to add to our last Report, the diseases being in many respects similar, or only altered, as might be expected, from the advance of spring, with improved temperature, and a drier atmosphere. The much talked-of typhus has almost disappeared, and would probably, by this time, have been overlooked, but for the convenience of the term, the convenience of nomenclature, and, perhaps, other conveniences, which delicacy prevents our enlarging upon. Phthisical patients have a respite, if we can call it such: however, they are easier. This has restored that flattering hope so characteristic of the disease; but which their sufferings, during the late changeable weather, had nearly stifled. A few rheumatisms occur in a mild form, and are easily subdued by brisk cathartics; in a still more chronic stage, the Reporter conceives he has found some specific effects from guaiacum. Hæmorrhage from all the emunctories have been frequent. Two patients, who never experienced it before, have had considerable hæmorrhage by the rectum; and one child, whilst changing his teeth, seems sympathetically attacked with hæmaturea: the quantity of blood, it should be observed, is probably less than it appears, as it is well known, that urine has the power of coagulating blood, by which the appearance of crasamentum is considerably increased.*

The uterine case, mentioned in our last, is going from bad to worse. On examination, *per vaginam*, the os tincæ is found agglutinated as in pregnancy; the uterus has ascended suddenly, after being considerably enlarged; and the alteration of the position has been attended with sensations similar (though in a higher degree) to what is felt by pregnant females. The patient is a widow more than sixty years of age. The discharge is somewhat increased, and occasionally streaked with blood.

Belladonna does not lose its reputation; but, we are glad to find another resource in tar, which we shall certainly keep in view, always keeping our motto in view—*Mille mali species, mille salutis erunt.*

---

* See a paper by Sir Everard Home, in the Philosophical Transactions.

### METEOROLOGICAL JOURNAL,

### By Messrs. William Harris and Co. 50, Holborn, London.

*From the 20th April to the 19th May, inclusive.*

| Day of Month. | Moon. | Rain gauge | Therm. | | | Barom. | | De Luc's Hygrom. Dry | | Damp | | Wind. | | Atmospheric Variation. | | |
|---|---|---|---|---|---|---|---|---|---|---|---|---|---|---|---|---|
| Apr. | | | | | | | | | | | | | | | | |
| 20 | ○ | | 47 | 52 | 39 | 29·75 | 29·73 | 0 | 0 | 7 | 12 | E | NNE | Fine | | |
| 21 | | | 45 | 51 | 41 | 29·70 | 29·75 | | | 9 | 12 | NE | E | Fine | | Clo. |
| 22 | | | 47 | 53 | 39 | 29·69 | 29·65 | | | 16 | 21 | ENE | E | Clo. | | Rain |
| 23 | | ,25 | 42 | 47 | 41 | 29·42 | 29·37 | | | 25 | 27 | E | E | Rain | | |
| 24 | | ,49 | 45 | 51 | 40 | 29·35 | 29·30 | | | 21 | 11 | ENE | NE | Clo. | Rain | |
| 25 | | ,31 | 42 | 49 | 44 | 29·30 | 29.30 | | | 10 | 10 | SE | SSW | Clo. | Rain | |
| 26 | | ,47 | 49 | 60 | 46 | 29·39 | 29·41 | | | 9 | 7 | E | E | Fine | | Light ning |
| 27 | ◑ | ,35 | 52 | 61 | 50 | 29·51 | 29·68 | | | 5 | 9 | SE | S | Rain | Fine | |
| 28 | | ,23 | 55 | 63 | 46 | 29·83 | 29·98 | | | 7 | 7 | SW | SW | Fine | | |
| 29 | | ,12 | 52 | 59 | 47 | 29·95 | 29·90 | | | 10 | 13 | S | ENE | Fine | | |
| 30 | | ,14 | 53 | 57 | 45 | 29·74 | 29·60 | | | 20 | 25 | NE | SSW | Clo. | Rain | Clo. |
| May | | | | | | | | | | | | | | | | |
| 1 | | | 49 | 55 | 40 | 29·73 | 29·91 | | | 15 | 20 | SW | S | Fine | | |
| 2 | | | 45 | 51 | 49 | 29·82 | 29·68 | | | 11 | 20 | E | NE | Show-ery | Fine | |
| 3 | | ,15 | 53 | 60 | 50 | 29·50 | 29·50 | | | 24 | 18 | E | S | Rain | Fine | |
| 4 | | ,21 | 54 | 63 | 51 | 29·50 | 29·52 | | | 15 | 17 | SW | W | Fine | | |
| 5 | ◐ | | 53 | 59 | 50 | 29·49 | 29·38 | | | 21 | 15 | SSE | ENE | Fine | | Rain |
| 6 | | ,10 | 54 | 61 | 50 | 29·29 | 29·33 | | | 17 | 21 | SW | S | Clo. | Rain | Fine |
| 7 | | ,11 | 54 | 63 | 49 | 29·40 | 29·47 | | | 20 | 17 | S | S | Clo. | Rain | Fine |
| 8 | | ,17 | 54 | 59 | 47 | 29·58 | 29·50 | | | 13 | 27 | S | NW | Clo. | Rain | |
| 9 | | ,20 | 53 | 57 | 49 | 29·69 | 29·75 | | | 25 | 27 | SSW | SSW | Fine | Rain | Show-ery |
| 10 | | ,19 | 52 | 59 | 49 | 29·86 | 29·83 | | | 25 | 20 | S | S | Fine | Clo. | Rain |
| 11 | | ,12 | 55 | 65 | 50 | 29·75 | 29·68 | | | 21 | 17 | S | S | Fine | | |
| 12 | | | 52 | 67 | 48 | 29·60 | 29·68 | | | 10 | 15 | S | SW | Fine | | |
| 13 | ○ | | 50 | 57 | 49 | 29·51 | 29·45 | | | 12 | 17 | S | S | Rain | Fine | |
| 14 | | ,13 | 52 | 58 | 48 | 29·42 | 29.52 | | | 15 | 21 | SE | SE | Rain | Show-ery | Fine |
| 15 | | ,18 | 55 | 60 | 49 | 29·51 | 29·50 | | | 7 | 12 | SE | SE | Fine | | |
| 16 | | | 55 | 63 | 49 | 29·54 | 29·65 | | | 15 | 17 | NE | NW | Fine | | |
| 17 | | | 53 | 59 | 49 | 29·70 | 29·79 | | | 15 | 20 | WNW | N | Clo. | | Rain |
| 18 | | ,11 | 56 | 64 | 49 | 29·88 | 29·95 | | | 15 | 15 | N | NE | Fine | | |
| 19 | | ,08 | 55 | 61 | 45 | 29.98 | 30·02 | | | 15 | 12 | N | NE | Clo. | Fine | |

The quantity of rain fallen in the month of April is 3 inches and 10-100ths.

